# Crystal structures of two isostructural bivalent metal *N*-benzoyl­glycinates

**DOI:** 10.1107/S2056989020009287

**Published:** 2020-07-10

**Authors:** Kedar U. Narvekar, Bikshandarkoil R. Srinivasan

**Affiliations:** a School of Chemical Sciences, Goa University PO, Goa 403206, India

**Keywords:** crystal structure, bivalent metal, *N*-benzoyl­glycine, μ_2_-bridging aqua ligand, isostructural, hydrogen bonding, inter­chain inter­action

## Abstract

The crystal structures of two coordination compounds of *N*-benzoyl­glycine, *viz. catena*-poly[[[di­aqua­bis­(*N*-benzoyl­glycinato)cobalt(II)]-μ-aqua] dihydrate], {[Co(C_9_H_8_NO_3_)_2_(H_2_O)_3_]·2H_2_O}_*n*_, **1**, and *catena*-poly[[[di­aqua­bis­(*N*-benzoyl­glycinato)nickel(II)]-μ-aqua] dihydrate], {[Ni(C_9_H_8_NO_3_)_2_(H_2_O)_3_]·2H_2_O}_*n*_, **2**, are described. The structures of **1** and **2** were reported previously [Morelock *et al.* (1979). *J. Am. Chem. Soc.* **101**, 4858–4866] and redetermined in this work to determine the H-atom coordinates. In the isostructural compounds, the central metal is located on an inversion centre and exhibits a distorted octa­hedral geometry. A pair of terminal aqua ligands disposed *trans* to each other and a pair of monodentate *N*-benzoyl­glycinate ligands form the square base and account for four of the six vertices of the octa­hedron. A μ_2_-bridging aqua ligand links the bivalent metals into one-dimensional chains extending along the *c-*axis direction. The one-dimensional chains stabilized by O—H⋯O hydrogen bonds are inter­linked by N—H⋯O and C—H⋯O hydrogen-bonding inter­actions.

## Abstract 2

The crystal structures of two coordination compounds of *N*-benzoyl­glycine, *viz. catena*-poly[[[di­aqua­bis­(*N*-benzoyl­glycinato)cobalt(II)]-μ-aqua] dihydrate], {[Co(C_9_H_8_NO_3_)_2_(H_2_O)_3_]·2H_2_O}_*n*_, and *catena*-poly[[[di­aqua­bis­(*N*-benzoyl­glycinato)nickel(II)]-μ-aqua] dihydrate], {[Ni(C_9_H_8_NO_3_)_2_(H_2_O)_3_]·2H_2_O}_*n*_, are described.

## Chemical context   

Hippuric acid known by other names such as *N*-benzoyl­glycine or benzoyl­amino­ethanoic acid or *N*-(benzene­carbon­yl)glycine is a derivative of glycine and is produced in metabolic processes (Pero, 2010 ▸). Hence the benzoyl-substituted glycine, namely *N*-benzoyl­glycine and its compounds, have been the subject of several investigations. The crystal structures of *N*-benzoyl­glycine and many of its derivatives are archived in the Cambridge Structural Database (CSD, version 5.40, update of September 2019; Groom *et al.*, 2016 ▸). Unlike *N*-benzoyl­glycine, which crystallizes in the non-centrosymmetric Sohncke space group *P*2_1_2_1_2_1_, a majority of its deriv­atives are centrosymmetric solids. In most of these compounds, *N*-benzoyl­glycine functions as a charge-balancing (*N*-benzoyl­glycinate) anion. In addition, the anion can also coordinate to a metal as observed in the title compounds. The *N*-benzoyl­glycinates of Co^II^ **1** and Ni^II^ **2** are some of the first examples of a series of α-amino acid compounds of the first-row transition-metal ions that exhibit low-dimensional magnetic properties (Morelock *et al.*, 1979 ▸). Based on a study of the visible spectra and the magnetic properties, compound **1** was shown to be a metamagnet and **2** an anti­ferromagnet.

**Figure 1:**
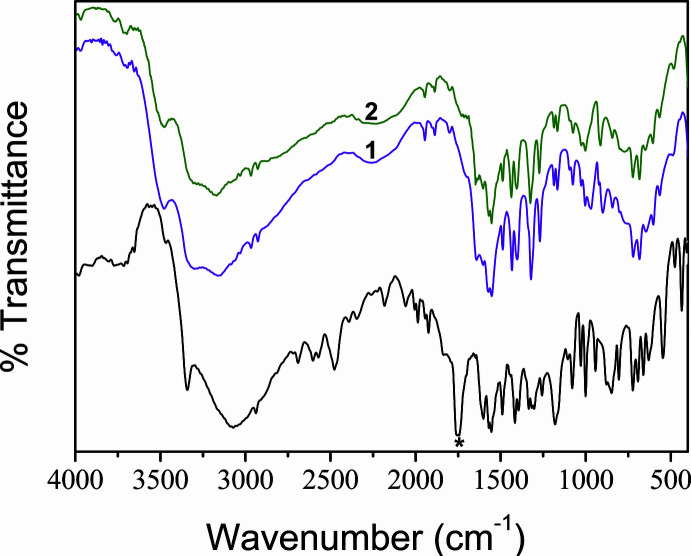
Infrared spectra of **1**, **2** and *N*-benzoyl­glycine (bottom). * corresponds to the signal for –COOH.

In the previous report, the title compounds **1** and **2** were prepared in an aqueous ethano­lic medium by the reaction of the sodium salt of hippuric acid with the corresponding bivalent metal perchlorate (Morelock *et al.* 1979 ▸). The polymeric structure of **1** and **2** due to aqua bridging was described, but the hydrogen-atom coordinates were not reported. *N*-Benzoyl­glycinates with a different stoichiometry represented by the formula *M*(C_9_H_8_NO_3_)_2_·6H_2_O (*M* = Co or Ni) are also known in the literature (Marcotrigiano & Pellacani, 1975 ▸). However, these were not structurally characterized. In the present work we have synthesized the title compounds by a direct acid–base reaction of cobalt carbonate (or nickel carbonate) with *N*-benzoyl­glycine (hippuric acid) to obtain [Co(H_2_O)_3_(C_9_H_8_NO_3_)_2_]·2H_2_O, **1**, and [Ni(H_2_O)_3_(C_9_H_8_NO_3_)_2_]·2H_2_O, **2**, respectively. The infrared spectra of both compounds are nearly identical, indicating similar structures. A comparison of the spectra of **1** and **2** with that of the free ligand (*N*-benzoyl­glycine) reveals notable changes in the profile of the spectra in the 3700–2750 cm^−1^ region. This can be explained by the presence of water mol­ecules in **1** and **2**, unlike in the free acid. *N*-Benzoyl­glycine exhibits a strong signal at ∼1743 cm^−1^ assignable for the –COOH vibration, which is shifted to lower energies in **1** and **2** due to deprotonation (Fig. 1 ▸). Despite a slightly different synthetic methodology, the product obtained by us is the same as evidenced by the structural details of **1** and **2**, which are in good agreement with the earlier work (Morelock *et al.* 1979 ▸) as shown below.
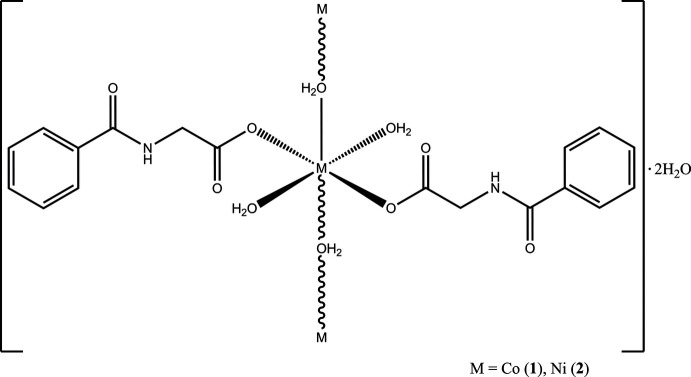


## Structural commentary   

**Figure 2:**
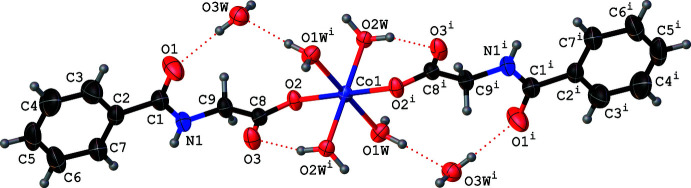
The mol­ecular structure of **1** showing the crystallographic labelling with displacement ellipsoids drawn at 50% probability level. Hydrogen atoms are drawn as spheres of arbitrary radius. Intra­molecular hydrogen bonds are shown as red dotted lines. Symmetry code: (i) 1 − *x*, 1 − *y*, 1 − *z*.

**Figure 3:**
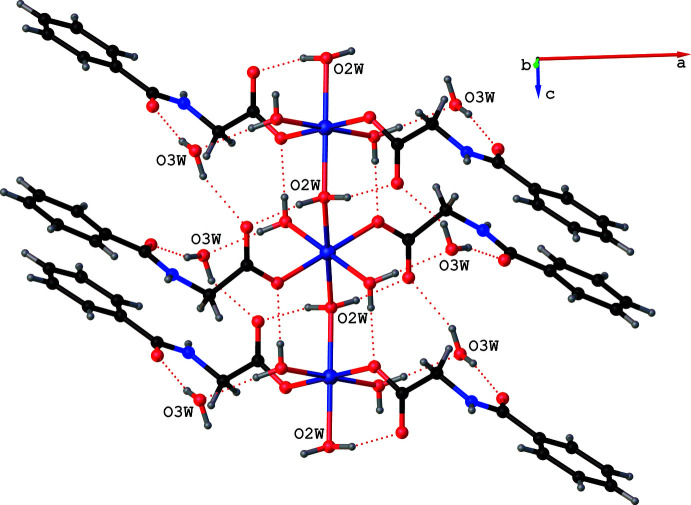
A portion of the one-dimensional chain formed by the bridging bidentate water mol­ecules (O2*W*), which extends the structure of **1** along the *c*-axis direction. The dotted red lines correspond to O—H⋯O hydrogen bonds.

The mol­ecular structure of the isostructural compounds [*M*(H_2_O)_3_(C_9_H_8_NO_3_)_2_]·2H_2_O (*M* = Co **1**, *M* = Ni **2**) is illus­trated in Fig. 2 ▸. Compounds **1** and **2** crystallize in the centrosymmetric monoclinic space group *C*2/*c* with the central cobalt (or nickel) ion located on an inversion centre. All of the atoms in both structures have been labelled so as to maintain parity for the ligand oxygen atoms and donor hydrogen and acceptor oxygen atoms in the hydrogen-bonding scheme. Other than the central metal, the structure consists of a unique terminal water (O1*W*), a unique monodentate *N*-benzoyl­glycinate (O2), a bridging aqua ligand (O2*W*) with the oxygen situated on a twofold axis and a non-ligated water (O3*W*), which constitute half of the formula unit of **1** or **2**. In view of the special position of the central metal, the other half is generated by the application of inversion symmetry. The geometric parameters of the *N*-benzoyl­glycinates are in the normal ranges and are in agreement with reported data (Natarajan *et al.*, 2007 ▸). The metal–oxygen bond distances (Tables 1 ▸ and 2 ▸) scatter in a very narrow range [2.0563 (15) to 2.1899 (9) Å in **1**; 2.029 (2) to 2.1450 (12) Å in **2**]. In both compounds, the carboxyl­ate oxygen (O2) of the *N*-benzoyl­glycinate makes the shortest *M*—O bond length while the longest *M*—O bond distance is observed for the bridging aqua ligand (O2*W*). Both compounds exhibit ideal values for the *trans* O—*M*—O bond angles while the *cis* O—*M*—O angles show a slight deviation [87.41 (6) to 92.59 (6)° in **1**; 87.27 (8) to 92.73 (8)° in **2**] indicating a slight distortion of the {*M*O_6_} octa­hedron (Tables 1 ▸ and 2 ▸). The difference Δ between the longest and the shortest *M*—O bonds can be considered as a measure of the distortion from ideal geometry and is 0.1336 (0.18) and 0.114 (0.12) Å for compounds **1** and **2**, respectively. The values in brackets are the difference Δ calculated from the reported bond distances of the earlier study. It is inter­esting to note that the same trend is observed with {CoO_6_} octa­hedron being slightly more distorted. The central metal exhibits hexa coordination and is bonded to two terminal aqua ligands (O1*W*, O1*W* ^i^) [symmetry code: (i) −*x* + 1, −*y* + 1, −*z* + 1] disposed *trans* to each other and two monodentate *N*-benzoyl­glycinate (O2, O2^i^) ligands accounting for the square base of the octa­hedron. The μ_2_-bridging binding mode of the aqua ligand (O2*W*) makes two axial bonds *trans* to each other completing the octa­hedral geometry around the central metal. The bridging binding mode results in the formation of a one-dimensional chain structure extending along the *c-*axis direction (Fig. 3 ▸). In the infinite chain, the observed *M*⋯*M* separations of 4.0015 (2) Å or 3.9492 (8) Å in **1** or **2**, respectively, are in very good agreement with the earlier work (Morelock *et al.* 1979 ▸). The *M*—O2*W*—*M* ^ii^ bond angle θ for **1** [symmetry code: (ii) −*x* + 1, *y*, −*z* + 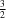] and **2** [symmetry code: (ii) −*x* + 1, *y*, −*z* + 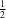] are 132.03 (11) and 134.02 (15)° for **1** and **2**, respectively, which follow the earlier trend with the reported θ values being 128.3 and 137.2° (Morelock *et al.* 1979 ▸). The Θ value is marginally higher for **2** and is accompanied by a shorter Ni1—O2*W* bond distance of 2.1450 (12) Å. The decreasing bond distance is attributed to increasing orbital overlap, explaining the larger superexchange in **2** leading to spin-pairing.

## Supra­molecular features   

**Figure 4:**
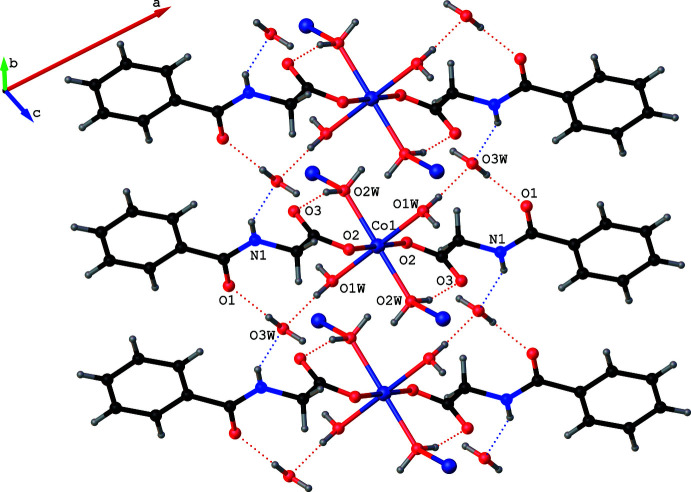
Non-ligated water (O3*W*) inter­links adjacent chains *via* N—H⋯O hydrogen bonds (shown as blue dotted lines). Intra­chain O—H⋯O hydrogen bonds are shown as red dotted lines. For clarity, the terminal ligands are displayed only for the metal in the middle of each chain.

**Figure 5:**
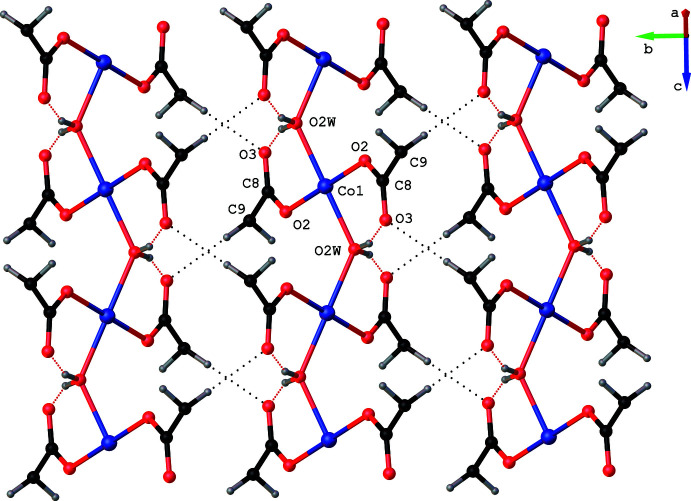
The C9—H9*B*⋯O3^iv^ inter­action (shown as black dotted lines) links the H9*B* atom in one chain with the O3 atom of a neighbouring chain. Intra­chain O—H⋯O hydrogen bonds are shown as red dotted lines. For clarity, only the –CH_2_—COO group of *N*-benzoyl­glycinate is displayed. The terminal aqua ligands and the non-ligated water are omitted.

The isostructural compounds **1** and **2** exhibit several non-covalent inter­actions, namely O—H⋯O, N—H⋯O and C—H⋯O hydrogen bonds (Tables 3 ▸ and 4 ▸) in their supra­molecular structures. All of the hydrogen atoms attached to the water mol­ecules, the hydrogen atom bonded to nitro­gen N1 and a hydrogen atom attached to the methyl­ene carbon C9 function as hydrogen donors and four of the six oxygen atoms, namely O1, O2, O3 and O3*W*, function as hydrogen acceptors. All of the O—H⋯O hydrogen bonds are intra­chain inter­actions (Fig. 3 ▸). The non-ligated water O3*W* inter­links adjacent chains with the aid of a single short N1—H1⋯O3*W* inter­action at H⋯*A* distances of 2.13 (3) and 2.04 (5) Å in **1** and **2**, respectively, accompanied by *D*—H⋯*A* angles of 149 (2) and 151 (4)° (Fig. 4 ▸). A short C9—H9*B*⋯O3^iv^ inter­action at a H⋯*A* distance 2.51 Å in **1** (2.50 Å in **2**) accompanied by *D*—H⋯*A* angle of 177.9° in **1**, (176.4° in **2**) links the H9*B* atom of a methyl­ene group of *N*-benzoyl­glycinate in one chain with the O3 atom of a symmetry-related *N*-benzoyl­glycinate in a neighboring chain functioning as a hydrogen acceptor (Fig. 5 ▸). These inter­chain hydrogen-bonding inter­actions serve to hold the chains together along the *b* axis, forming a layer of chains in the *bc* plane. Thus, the findings of our present study once again support the original findings, namely compounds **1** and **2** are unique examples of psuedo one-dimensional (1D) magnetic materials in which three-dimensional magnetic ordering was predicted not to occur until *T* → 0 K. In addition to the hydrogen-bonding inter­actions, **1** and **2** exhibit π–π stacking inter­actions (Hunter & Sanders, 1990 ▸). For the analysis of short ring inter­actions, the program *PLATON* (Spek, 2020 ▸) was used. The ring centroid–centroid distances (*Cg*⋯*Cg*) between the adjacent benzene rings in **1** and **2** are found to be 4.0435 (2) and 3.9807 (5) Å, respectively. It has been reported that stacking inter­actions can exist at very long *Cg*⋯*Cg* distances of up to 7 Å (Ninković *et al.*, 2011 ▸). Hence, the observed *Cg*⋯*Cg* distances can be attributed to the π–π stacking of the benzene rings.

## Database survey   

The Cambridge Structural Database (CSD, version 5.40, update of September 2019; Groom *et al.*, 2016 ▸) lists several structurally characterized organic and metal–organic compounds of *N*-benzoyl­glycine. Since the first report on the crystal structure of *N*-benzoyl­glycine (Ringertz, 1971 ▸), several compounds of *N*-benzoyl­glycine have been structurally characterized. Excepting an example of a 1:1 co-crystal of *N*-benzoyl­glycine, namely glibenclamide hippuric acid (Goyal *et al.*, 2017 ▸), the structures of thirty two compounds containing the monoanionic *N*-benzoyl­glycinate were retrieved from the CSD (Groom *et al.*, 2016 ▸). Three of these do not contain any metal and are charge-balanced by organic cations (Görbitz & Sagstuen, 2004 ▸; Chadha *et al.*, 2016 ▸; John *et al.*, 2018 ▸). Of the twenty nine examples of *N*-benzoyl­glycinates with metal–organic cations, eight contain bivalent metal (Table 5 ▸) and aqua ligands. In this work, a comparative study of bivalent metal *N*-benzoyl­glycinates containing only aqua ligands has been undertaken. It is inter­esting to note that all of these compounds contain coordinated water mol­ecules. In this list of compounds, excepting the *N*-benzoyl­glycinate of Zn^II^ (Grewe *et al.*, 1982 ▸), the rest are all centrosymmetric. In all eight compounds, the *N*-benzoyl­glycinate coordinates to the metal only through the carboxyl­ate oxygen atoms. In five of these, including the title compounds, *N*-benzoyl­glycinate functions as a monodentate ligand. The bridging binding mode in the *N*-benzoyl­glycinates of Ca^II^ (Jisha *et al.*, 2010 ▸), Ba^II^ (Natarajan *et al.* 2007 ▸), Cu^II^ (Brown & Trefonas, 1973 ▸) and Pb^II^ (Battistuzzi *et al.*, 1996 ▸) can explain the polymeric nature of these compounds, excepting the Cu^II^ which is a dimer. The structure of the dimeric copper compound (Refcode CUHIPT; Brown & Trefonas, 1973 ▸) contains both a monodentate as well as a monoatomic bridging *N*-benzoyl­glycinate. It is inter­esting to note that the dinuclear Cu^II^ compound of *N*-benzoyl­glycine does not adopt the paddle-wheel structure. The *N*-benzoyl­glycinate of Fe^II^ (Morelock *et al.*, 1982 ▸) is also isostructural with the title compounds and is a 1D polymer. It is inter­esting to note that in the three isostructural *N*-benzoyl­glycinates of 3*d* metals, an aqua ligand functions as a bridging ligand to extend the structure, and not the *N*-benzoyl­glycinate.

## Synthesis and crystallization   

For the synthesis of **1**, *N*-benzoyl­glycine (1.792 g, 10 mmol) taken in distilled water (50 mL) was heated with stirring to obtain a clear solution. Into this, CoCO_3_ (0.595 g, 5 mmol) was added in small portions. Brisk effervescence was observed accompanied by the dissolution of the insoluble carbonate, resulting in a pink-coloured solution. When most of the carbonate had dissolved, a small amount (∼25 mg) of the carbonate was added and the heating continued for a further hour. The hot reaction mixture was filtered and the clear pink filtrate was left undisturbed for crystallization. The crystals obtained after a few days were isolated by filtration and dried in air, yield = 90%. A similar procedure was employed for **2** using nickel carbonate instead of cobalt carbonate and the filtrate obtained was light green. Crystals were isolated as before, yield = 80%.

## Refinement   

Crystal data, data collection and structure refinement details are summarized in Table 6 ▸. O- and N-bound H atoms were freely refined. C-bound hydrogen atoms were placed at calculated positions C—H = 0.93–0.97 Å) and refined isotropically [*U*_iso_(H) = 1.2*U*_eq_(C). using a riding-atom model.

## Supplementary Material

Crystal structure: contains datablock(s) 1, 2. DOI: 10.1107/S2056989020009287/ex2034sup1.cif

Structure factors: contains datablock(s) 1. DOI: 10.1107/S2056989020009287/ex20341sup2.hkl

Structure factors: contains datablock(s) 2. DOI: 10.1107/S2056989020009287/ex20342sup3.hkl

CCDC references: 2014672, 2014671

Additional supporting information:  crystallographic information; 3D view; checkCIF report

## Figures and Tables

**Table 1:** Selected geometric parameters (Å, °) for **1** [Chem scheme1]

Co1—O2	2.0563 (15)	Co1—O1*W* ^i^	2.0622 (17)
Co1—O2^i^	2.0563 (15)	Co1—O2*W* ^i^	2.1899 (9)
Co1—O1*W*	2.0622 (17)	Co1—O2*W*	2.1899 (9)
			
O2—Co1—O2^i^	180.0	O1*W*—Co1—O2*W* ^i^	90.55 (7)
O2—Co1—O1*W*	89.40 (7)	O1*W* ^i^—Co1—O2*W* ^i^	89.45 (7)
O2^i^—Co1—O1*W*	90.60 (7)	O2—Co1—O2*W*	87.41 (6)
O2—Co1—O1*W* ^i^	90.60 (7)	O2^i^—Co1—O2*W*	92.59 (6)
O2^i^—Co1—O1*W* ^i^	89.40 (7)	O1*W*—Co1—O2*W*	89.45 (7)
O1*W*—Co1—O1*W* ^i^	180.0	O1*W* ^i^—Co1—O2*W*	90.55 (7)
O2—Co1—O2*W* ^i^	92.59 (6)	O2*W* ^i^—Co1—O2*W*	180.0
O2^i^—Co1—O2*W* ^i^	87.41 (6)	Co1^ii^—O2*W*—Co1	132.03 (11)

Symmetry codes: (i) 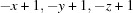; (ii) 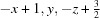.

**Table 2:** Selected geometric parameters (Å, °) for **2** [Chem scheme1]

Ni1—O2^i^	2.029 (2)	Ni1—O1*W*	2.041 (2)
Ni1—O2	2.029 (2)	Ni1—O2*W* ^i^	2.1450 (12)
Ni1—O1*W* ^i^	2.041 (2)	Ni1—O2*W*	2.1450 (12)
			
O2^i^—Ni1—O2	180.00 (12)	O1*W* ^i^—Ni1—O2*W* ^i^	89.87 (8)
O2^i^—Ni1—O1*W* ^i^	88.72 (10)	O1*W*—Ni1—O2*W* ^i^	90.13 (8)
O2—Ni1—O1*W* ^i^	91.28 (10)	O2^i^—Ni1—O2*W*	92.73 (8)
O2^i^—Ni1—O1*W*	91.28 (10)	O2—Ni1—O2*W*	87.27 (8)
O2—Ni1—O1*W*	88.72 (10)	O1*W* ^i^—Ni1—O2*W*	90.13 (8)
O1*W* ^i^—Ni1—O1*W*	180.0	O1*W*—Ni1—O2*W*	89.87 (8)
O2^i^—Ni1—O2*W* ^i^	87.27 (8)	O2*W* ^i^—Ni1—O2*W*	180.0
O2—Ni1—O2*W* ^i^	92.73 (8)	Ni1—O2*W*—Ni1^ii^	134.02 (15)

Symmetry codes: (i) 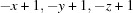; (ii) 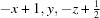.

**Table 3:** Hydrogen-bond geometry (Å, °) for **1** [Chem scheme1]

*D*—H⋯*A*	*D*—H	H⋯*A*	*D*⋯*A*	*D*—H⋯*A*
O1*W*—H1*WA*⋯O2^ii^	0.78 (3)	1.93 (3)	2.714 (2)	176 (3)
O1*W*—H1*WB*⋯O3*W* ^i^	0.85 (3)	1.93 (3)	2.780 (3)	178 (3)
O2*W*—H2*W*⋯O3^iii^	0.88 (3)	1.80 (3)	2.6576 (18)	163 (3)
C9—H9*B*⋯O3^iv^	0.97	2.51	3.481 (3)	178
N1—H1⋯O3*W* ^v^	0.83 (3)	2.13 (3)	2.880 (3)	149 (2)
O3*W*—H3*WA*⋯O1	0.83 (4)	1.90 (4)	2.708 (3)	164 (3)
O3*W*—H3*WB*⋯O3^iii^	0.84 (3)	2.12 (3)	2.873 (3)	149 (3)

Symmetry codes: (i) 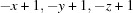; (ii) 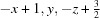; (iii) 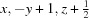; (iv) 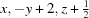; (v) 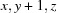.

**Table 4:** Hydrogen-bond geometry (Å, °) for **2** [Chem scheme1]

*D*—H⋯*A*	*D*—H	H⋯*A*	*D*⋯*A*	*D*—H⋯*A*
O1*W*—H1*WA*⋯O2^ii^	0.90 (5)	1.82 (5)	2.726 (3)	174 (5)
O1*W*—H1*WB*⋯O3*W* ^i^	0.81 (5)	1.97 (5)	2.783 (4)	176 (5)
O2*W*—H2*W*⋯O3^i^	0.77 (4)	1.88 (4)	2.634 (3)	167 (4)
C9—H9*B*⋯O3^iii^	0.97	2.50	3.465 (4)	176
N1—H1⋯O3*W* ^iv^	0.93 (5)	2.04 (5)	2.888 (4)	151 (4)
O3*W*—H3*WB*⋯O3^v^	0.86 (6)	2.10 (6)	2.858 (4)	147 (5)
O3*W*—H3*WA*⋯O1	0.81 (9)	1.92 (9)	2.697 (4)	159 (8)

Symmetry codes: (i) 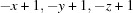; (ii) 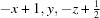; (iii) 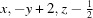; (iv) 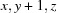; (v) 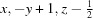.

**Table 5:** Comparative structural chemistry of bivalent metal *N*-benzoyl­glycinates CN = coordination number of metal, C_9_H_9_NO_3_ = *N*-benzoyl­glycine, C_9_H_8_NO_3_ = *N*-benzoyl­glycinate.

Compound	Space group	CN	Binding mode	Dimensionality	Refcode
C_9_H_9_NO_3_	*P*2_1_2_1_2_1_	-	-	monomer	HIPPAC
[Ca(H_2_O)_2_(C_9_H_8_NO_3_)_2_]·H_2_O	*P*2_1_/*c*	8	μ_2_-tridentate	one-dimensional	ANEDON
[Ba_2_(H_2_O)_3_(C_9_H_8_NO_3_)_4_]	*P* 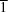	9, 10	μ_3_-tridentate, μ_3_-tetra­dentate	two-dimensional	HIFFIM
[Fe(H_2_O)_3_(C_9_H_8_NO_3_)_2_]·2H_2_O	*C*2/*c*	6	monodentate	one-dimensional	BITDAJ
[Co(H_2_O)_3_(C_9_H_8_NO_3_)_2_]·2H_2_O	*C*2/*c*	6	monodentate	one-dimensional	COHIPP10, this work
[Ni(H_2_O)_3_(C_9_H_8_NO_3_)_2_]·2H_2_O	*C*2/*c*	6	monodentate	one-dimensional	ANIHIP, this work
[Cu_2_(H_2_O)_4_(C_9_H_8_NO_3_)_4_]·2H_2_O	*P*2_1_/*c*	5, 5	monodentate, μ_2_-monoatomic	dimer	CUHIPT
[Zn(H_2_O)_3_(C_9_H_8_NO_3_)_2_]·2H_2_O	*P*1	5	monodentate	monomer	BIZFUL
[Pb(H_2_O)_2_(C_9_H_8_NO_3_)_2_]·2H_2_O	*C*2/*c*	8	μ_2_-tridentate	one-dimensional	TEZMOA

References: HIPPAC: Ringertz (1971 ▸); ANEDON: Jisha *et al.* (2010 ▸); HIFFIM: Natarajan *et al.* (2007 ▸); BITDAJ: Morelock *et al.* (1982 ▸); COHIPP10: Morelock *et al.* (1979 ▸); CUHIPT: Brown & Trefonas (1973 ▸); BIZFUL: Grewe *et al.* (1982 ▸); TEZMOA: Battistuzzi *et al.* (1996 ▸).

**Table 6:** Experimental details

	**1**	**2**
Crystal data		
Chemical formula	[Co(C_9_H_8_NO_3_)_2_(H_2_O)_3_]·2H_2_O	[Ni(C_9_H_8_NO_3_)_2_(H_2_O)_3_]·2H_2_O
*M*_r_	505.34	505.12
Crystal system, space group	Monoclinic, *C*2/*c*	Monoclinic, *C*2/*c*
Temperature (K)	293	293
*a*, *b*, *c* (Å)	40.843 (2), 6.9072 (4), 8.0031 (4)	40.884 (4), 6.9438 (8), 7.8983 (8)
β (°)	91.891 (2)	91.900 (2)
*V* (Å^3^)	2256.6 (2)	2241.0 (4)
*Z*	4	4
Radiation type	Mo *K*α	Mo *K*α
μ (mm^−1^)	0.82	0.93
Crystal size (mm)	0.35 × 0.27 × 0.04	0.29 × 0.24 × 0.05

Data collection		
Diffractometer	Bruker D8 Quest Eco	Bruker D8 Quest Eco
Absorption correction	Numerical (*SADABS*; Krause *et al.*, 2015 ▸)	Numerical (*SADABS*; Krause *et al.*, 2015 ▸)
*T*_min_, *T*_max_	0.610, 0.746	0.608, 0.746
No. of measured, independent and observed [*I* > 2σ(*I*)] reflections	15061, 2799, 2070	17095, 3392, 2995
*R*_int_	0.046	0.036
(sin θ/λ)_max_ (Å^−1^)	0.666	0.714

Refinement		
*R*[*F* ^2^ > 2σ(*F* ^2^)], *wR*(*F* ^2^), *S*	0.037, 0.086, 1.08	0.056, 0.151, 1.15
No. of reflections	2799	3392
No. of parameters	171	174
H-atom treatment	H atoms treated by a mixture of independent and constrained refinement	H atoms treated by a mixture of independent and constrained refinement
Δρ_max_, Δρ_min_ (e Å^−3^)	0.39, −0.43	1.24, −0.76

Computer programs: *APEX3* and *SAINT* (Bruker, 2019 ▸), *SHELXT2014/5* (Sheldrick, 2015*a* ▸), *SHELXL2018/3* (Sheldrick, 2015*b* ▸), *OLEX2* (Dolomanov *et al.*, 2009 ▸), *shelXle* (Hübschle *et al.*, 2011 ▸) and *publCIF* (Westrip, 2010 ▸).

## supplementary crystallographic information

### catena-Poly[[[diaquabis(N-benzoylglycinato)cobalt(II)]-µ-aqua] dihydrate] (1) Crystal data

**Table:**

[Co(C_9_H_8_NO_3_)_2_(H_2_O)_3_]·2H_2_O	*F*(000) = 1052
*M**_r_* = 505.34	*D*_x_ = 1.487 Mg m^−^^3^
Monoclinic, *C*2/*c*	Mo *K*α radiation, λ = 0.71073 Å
*a* = 40.843 (2) Å	Cell parameters from 4616 reflections
*b* = 6.9072 (4) Å	θ = 3.0–28.0°
*c* = 8.0031 (4) Å	µ = 0.82 mm^−^^1^
β = 91.891 (2)°	*T* = 293 K
*V* = 2256.6 (2) Å^3^	Plate, pink
*Z* = 4	0.35 × 0.27 × 0.04 mm

### catena-Poly[[[diaquabis(N-benzoylglycinato)cobalt(II)]-µ-aqua] dihydrate] (1) Data collection

**Table:**

Bruker D8 Quest Eco diffractometer	2070 reflections with *I* > 2σ(*I*)
Radiation source: Sealed Tube	*R*_int_ = 0.046
φ and ω scans	θ_max_ = 28.3°, θ_min_ = 3.0°
Absorption correction: numerical (SADABS; Krause *et al.*, 2015)	*h* = −54→54
*T*_min_ = 0.610, *T*_max_ = 0.746	*k* = −9→9
15061 measured reflections	*l* = −10→10
2799 independent reflections	

### catena-Poly[[[diaquabis(N-benzoylglycinato)cobalt(II)]-µ-aqua] dihydrate] (1) Refinement

**Table:**

Refinement on *F*^2^	0 restraints
Least-squares matrix: full	Hydrogen site location: mixed
*R*[*F*^2^ > 2σ(*F*^2^)] = 0.037	H atoms treated by a mixture of independent and constrained refinement
*wR*(*F*^2^) = 0.086	*w* = 1/[σ^2^(*F*_o_^2^) + (0.0214*P*)^2^ + 3.6234*P*] where *P* = (*F*_o_^2^ + 2*F*_c_^2^)/3
*S* = 1.08	(Δ/σ)_max_ < 0.001
2799 reflections	Δρ_max_ = 0.39 e Å^−^^3^
171 parameters	Δρ_min_ = −0.42 e Å^−^^3^

### catena-Poly[[[diaquabis(N-benzoylglycinato)cobalt(II)]-µ-aqua] dihydrate] (1) Special details

**Table:**

Geometry. All esds (except the esd in the dihedral angle between two l.s. planes) are estimated using the full covariance matrix. The cell esds are taken into account individually in the estimation of esds in distances, angles and torsion angles; correlations between esds in cell parameters are only used when they are defined by crystal symmetry. An approximate (isotropic) treatment of cell esds is used for estimating esds involving l.s. planes.
Refinement. Suitable single crystals were selected under a polarizing microscope in HR2-643 parabar 10312 oil from Hampton Research. The crystal was mounted on a 20 micron 0.4–0.5 mm HR4-953 Mounted Cryoloops loop from Hampton Research and transferred to the Bruker D8 Quest Eco diffractometer. Reflections harvested from two sets of 12, 0.5^ο^φ scans were used to determine unit-cell parameters and was used to determine the data-collection strategy. Unit-cell parameters were refined using reflections harvested from the data collection. The frames were integrated with the Bruker SAINT software package using a narrow-frame algorithm. The integration of the data using a monoclinic unit cell yielded a total of 15061 reflections to a maximum θ angle of 28.26° (0.75 Å resolution) for 1 and 17095 reflections to a maximum θ angle of 30.52° (0.70 Å resolution) for 2. All data were corrected for Lorentz and polarization effects and subsequently scaled. A numerical absorption correction was performed by *SADABS* (Krause *et al.*, 2015). The space group was determined and the structures were solved using the intrinsic phasing method (Bruker, 2019; Sheldrick, 2008). The structures were refined in *APEX3* v2019.1-0 by *SHELXL* (Sheldrick, 2015).

### catena-Poly[[[diaquabis(N-benzoylglycinato)cobalt(II)]-µ-aqua] dihydrate] (1) Fractional atomic coordinates and isotropic or equivalent isotropic displacement parameters (Å^2^)

**Table:**

	*x*	*y*	*z*	*U*_iso_*/*U*_eq_	
Co1	0.500000	0.500000	0.500000	0.02504 (12)	
O1W	0.53443 (4)	0.6984 (3)	0.5847 (2)	0.0359 (4)	
H1WA	0.5349 (7)	0.699 (4)	0.682 (4)	0.058 (10)*	
H1WB	0.5538 (7)	0.690 (4)	0.550 (3)	0.050 (9)*	
O2W	0.500000	0.3711 (3)	0.750000	0.0274 (5)	
H2W	0.4817 (7)	0.305 (4)	0.761 (4)	0.072 (10)*	
O2	0.46352 (4)	0.6817 (2)	0.57632 (18)	0.0327 (4)	
O3	0.44216 (4)	0.7725 (3)	0.32901 (18)	0.0397 (4)	
C8	0.44162 (5)	0.7613 (3)	0.4833 (3)	0.0283 (5)	
C9	0.41364 (5)	0.8469 (4)	0.5790 (3)	0.0334 (5)	
H9A	0.405396	0.748241	0.652987	0.040*	
H9B	0.422184	0.951677	0.648337	0.040*	
N1	0.38658 (5)	0.9200 (3)	0.4767 (3)	0.0349 (5)	
H1	0.3849 (6)	1.039 (4)	0.469 (3)	0.037 (8)*	
O1	0.36670 (5)	0.6242 (3)	0.4197 (3)	0.0629 (6)	
C1	0.36437 (6)	0.8018 (4)	0.4068 (3)	0.0388 (6)	
C2	0.33558 (6)	0.8888 (4)	0.3134 (3)	0.0385 (6)	
C3	0.31275 (8)	0.7639 (5)	0.2456 (4)	0.0642 (9)	
H3	0.316051	0.631037	0.255105	0.077*	
C4	0.28480 (8)	0.8330 (6)	0.1628 (4)	0.0752 (11)	
H4	0.269374	0.746529	0.118400	0.090*	
C5	0.27986 (7)	1.0263 (6)	0.1465 (4)	0.0667 (10)	
H5	0.261079	1.073072	0.091414	0.080*	
C6	0.30260 (7)	1.1508 (5)	0.2113 (4)	0.0647 (9)	
H6	0.299329	1.283351	0.198759	0.078*	
C7	0.33062 (7)	1.0849 (4)	0.2959 (4)	0.0499 (7)	
H7	0.345894	1.172283	0.340250	0.060*	
O3W	0.40263 (5)	0.3206 (3)	0.5364 (2)	0.0412 (4)	
H3WA	0.3916 (8)	0.420 (6)	0.520 (4)	0.081 (13)*	
H3WB	0.4083 (8)	0.319 (5)	0.639 (4)	0.074 (11)*	

### catena-Poly[[[diaquabis(N-benzoylglycinato)cobalt(II)]-µ-aqua] dihydrate] (1) Atomic displacement parameters (Å^2^)

**Table:**

	*U*^11^	*U*^22^	*U*^33^	*U*^12^	*U*^13^	*U*^23^
Co1	0.0247 (2)	0.0338 (2)	0.01649 (18)	0.0018 (2)	−0.00142 (13)	0.00037 (17)
O1W	0.0350 (10)	0.0495 (11)	0.0230 (9)	−0.0071 (8)	−0.0003 (7)	−0.0027 (8)
O2W	0.0294 (12)	0.0368 (13)	0.0159 (10)	0.000	0.0003 (8)	0.000
O2	0.0315 (8)	0.0446 (10)	0.0216 (7)	0.0107 (7)	−0.0029 (6)	−0.0006 (7)
O3	0.0398 (9)	0.0569 (11)	0.0222 (8)	0.0138 (9)	−0.0022 (7)	0.0012 (7)
C8	0.0303 (11)	0.0301 (12)	0.0241 (10)	0.0036 (10)	−0.0026 (8)	−0.0016 (9)
C9	0.0319 (12)	0.0397 (13)	0.0283 (11)	0.0086 (10)	−0.0033 (9)	−0.0052 (10)
N1	0.0309 (11)	0.0339 (12)	0.0396 (12)	0.0079 (9)	−0.0028 (8)	−0.0026 (9)
O1	0.0679 (14)	0.0367 (11)	0.0820 (16)	0.0057 (10)	−0.0293 (11)	−0.0015 (10)
C1	0.0371 (13)	0.0404 (14)	0.0385 (13)	0.0039 (12)	−0.0032 (10)	−0.0024 (11)
C2	0.0320 (13)	0.0474 (15)	0.0359 (13)	0.0030 (12)	−0.0002 (10)	0.0014 (11)
C3	0.0579 (19)	0.060 (2)	0.072 (2)	−0.0079 (16)	−0.0251 (16)	0.0049 (16)
C4	0.0534 (19)	0.097 (3)	0.073 (2)	−0.014 (2)	−0.0290 (17)	0.007 (2)
C5	0.0393 (15)	0.105 (3)	0.0552 (19)	0.0147 (19)	−0.0096 (13)	0.0112 (19)
C6	0.0544 (19)	0.070 (2)	0.069 (2)	0.0216 (17)	−0.0092 (16)	0.0097 (17)
C7	0.0415 (15)	0.0540 (17)	0.0539 (17)	0.0074 (14)	−0.0037 (12)	0.0005 (14)
O3W	0.0454 (11)	0.0412 (11)	0.0368 (11)	0.0000 (9)	−0.0036 (8)	0.0005 (8)

### catena-Poly[[[diaquabis(N-benzoylglycinato)cobalt(II)]-µ-aqua] dihydrate] (1) Geometric parameters (Å, º)

**Table:**

Co1—O2	2.0563 (15)	N1—H1	0.83 (3)
Co1—O2^i^	2.0563 (15)	O1—C1	1.235 (3)
Co1—O1W	2.0622 (17)	C1—C2	1.498 (3)
Co1—O1W^i^	2.0622 (17)	C2—C3	1.370 (4)
Co1—O2W^i^	2.1899 (9)	C2—C7	1.376 (4)
Co1—O2W	2.1899 (9)	C3—C4	1.386 (4)
O1W—H1WA	0.78 (3)	C3—H3	0.9300
O1W—H1WB	0.85 (3)	C4—C5	1.357 (5)
O2W—H2W	0.88 (3)	C4—H4	0.9300
O2W—H2W^ii^	0.88 (3)	C5—C6	1.356 (5)
O2—C8	1.270 (2)	C5—H5	0.9300
O3—C8	1.238 (2)	C6—C7	1.387 (4)
C8—C9	1.517 (3)	C6—H6	0.9300
C9—N1	1.445 (3)	C7—H7	0.9300
C9—H9A	0.9700	O3W—H3WA	0.83 (4)
C9—H9B	0.9700	O3W—H3WB	0.84 (3)
N1—C1	1.330 (3)		
			
O2—Co1—O2^i^	180.0	C8—C9—H9A	108.5
O2—Co1—O1W	89.40 (7)	N1—C9—H9B	108.5
O2^i^—Co1—O1W	90.60 (7)	C8—C9—H9B	108.5
O2—Co1—O1W^i^	90.60 (7)	H9A—C9—H9B	107.5
O2^i^—Co1—O1W^i^	89.40 (7)	C1—N1—C9	121.5 (2)
O1W—Co1—O1W^i^	180.0	C1—N1—H1	121.7 (18)
O2—Co1—O2W^i^	92.59 (6)	C9—N1—H1	116.7 (18)
O2^i^—Co1—O2W^i^	87.41 (6)	O1—C1—N1	121.7 (2)
O1W—Co1—O2W^i^	90.55 (7)	O1—C1—C2	119.8 (2)
O1W^i^—Co1—O2W^i^	89.45 (7)	N1—C1—C2	118.5 (2)
O2—Co1—O2W	87.41 (6)	C3—C2—C7	118.9 (3)
O2^i^—Co1—O2W	92.59 (6)	C3—C2—C1	117.3 (2)
O1W—Co1—O2W	89.45 (7)	C7—C2—C1	123.8 (2)
O1W^i^—Co1—O2W	90.55 (7)	C2—C3—C4	120.8 (3)
O2W^i^—Co1—O2W	180.0	C2—C3—H3	119.6
Co1—O1W—H1WA	109 (2)	C4—C3—H3	119.6
Co1—O1W—H1WB	118.5 (19)	C5—C4—C3	120.2 (3)
H1WA—O1W—H1WB	110 (3)	C5—C4—H4	119.9
Co1^ii^—O2W—Co1	132.03 (11)	C3—C4—H4	119.9
Co1^ii^—O2W—H2W	95 (2)	C6—C5—C4	119.3 (3)
Co1—O2W—H2W	109 (2)	C6—C5—H5	120.3
Co1^ii^—O2W—H2W^ii^	109 (2)	C4—C5—H5	120.3
Co1—O2W—H2W^ii^	95 (2)	C5—C6—C7	121.5 (3)
H2W—O2W—H2W^ii^	118 (4)	C5—C6—H6	119.3
C8—O2—Co1	126.39 (14)	C7—C6—H6	119.3
O3—C8—O2	125.2 (2)	C2—C7—C6	119.3 (3)
O3—C8—C9	121.20 (19)	C2—C7—H7	120.4
O2—C8—C9	113.61 (18)	C6—C7—H7	120.4
N1—C9—C8	115.14 (18)	H3WA—O3W—H3WB	108 (3)
N1—C9—H9A	108.5		
			
Co1—O2—C8—O3	−14.5 (3)	N1—C1—C2—C7	−0.2 (4)
Co1—O2—C8—C9	166.03 (14)	C7—C2—C3—C4	−1.1 (5)
O3—C8—C9—N1	6.9 (3)	C1—C2—C3—C4	177.5 (3)
O2—C8—C9—N1	−173.6 (2)	C2—C3—C4—C5	0.7 (6)
C8—C9—N1—C1	78.1 (3)	C3—C4—C5—C6	0.3 (6)
C9—N1—C1—O1	−3.4 (4)	C4—C5—C6—C7	−0.8 (5)
C9—N1—C1—C2	175.3 (2)	C3—C2—C7—C6	0.5 (4)
O1—C1—C2—C3	0.0 (4)	C1—C2—C7—C6	−178.0 (3)
N1—C1—C2—C3	−178.7 (3)	C5—C6—C7—C2	0.5 (5)
O1—C1—C2—C7	178.5 (3)		

Symmetry codes: (i) −*x*+1, −*y*+1, −*z*+1; (ii) −*x*+1, *y*, −*z*+3/2.

### catena-Poly[[[diaquabis(N-benzoylglycinato)cobalt(II)]-µ-aqua] dihydrate] (1) Hydrogen-bond geometry (Å, º)

**Table:**

*D*—H···*A*	*D*—H	H···*A*	*D*···*A*	*D*—H···*A*
O1*W*—H1*WA*···O2^ii^	0.78 (3)	1.93 (3)	2.714 (2)	176 (3)
O1*W*—H1*WB*···O3*W*^i^	0.85 (3)	1.93 (3)	2.780 (3)	178 (3)
O2*W*—H2*W*···O3^iii^	0.88 (3)	1.80 (3)	2.6576 (18)	163 (3)
C9—H9*B*···O3^iv^	0.97	2.51	3.481 (3)	178
N1—H1···O3*W*^v^	0.83 (3)	2.13 (3)	2.880 (3)	149 (2)
O3*W*—H3*WA*···O1	0.83 (4)	1.90 (4)	2.708 (3)	164 (3)
O3*W*—H3*WB*···O3^iii^	0.84 (3)	2.12 (3)	2.873 (3)	149 (3)

Symmetry codes: (i) −*x*+1, −*y*+1, −*z*+1; (ii) −*x*+1, *y*, −*z*+3/2; (iii) *x*, −*y*+1, *z*+1/2; (iv) *x*, −*y*+2, *z*+1/2; (v) *x*, *y*+1, *z*.

### catena-Poly[[[diaquabis(N-benzoylglycinato)nickel(II)]-µ-aqua] dihydrate] (2) Crystal data

**Table:**

[Ni(C_9_H_8_NO_3_)_2_(H_2_O)_3_]·2H_2_O	*F*(000) = 1056
*M**_r_* = 505.12	*D*_x_ = 1.497 Mg m^−^^3^
Monoclinic, *C*2/*c*	Mo *K*α radiation, λ = 0.71073 Å
*a* = 40.884 (4) Å	Cell parameters from 8047 reflections
*b* = 6.9438 (8) Å	θ = 3.0–30.5°
*c* = 7.8983 (8) Å	µ = 0.93 mm^−^^1^
β = 91.900 (2)°	*T* = 293 K
*V* = 2241.0 (4) Å^3^	Plate, green
*Z* = 4	0.29 × 0.24 × 0.05 mm

### catena-Poly[[[diaquabis(N-benzoylglycinato)nickel(II)]-µ-aqua] dihydrate] (2) Data collection

**Table:**

Bruker D8 Quest Eco diffractometer	2995 reflections with *I* > 2σ(*I*)
Radiation source: Sealed Tube	*R*_int_ = 0.036
φ and ω scans	θ_max_ = 30.5°, θ_min_ = 3.0°
Absorption correction: numerical (SADABS; Krause *et al.*, 2015)	*h* = −58→58
*T*_min_ = 0.608, *T*_max_ = 0.746	*k* = −9→9
17095 measured reflections	*l* = −11→11
3392 independent reflections	

### catena-Poly[[[diaquabis(N-benzoylglycinato)nickel(II)]-µ-aqua] dihydrate] (2) Refinement

**Table:**

Refinement on *F*^2^	0 restraints
Least-squares matrix: full	Hydrogen site location: mixed
*R*[*F*^2^ > 2σ(*F*^2^)] = 0.056	H atoms treated by a mixture of independent and constrained refinement
*wR*(*F*^2^) = 0.151	*w* = 1/[σ^2^(*F*_o_^2^) + (0.0534*P*)^2^ + 10.915*P*] where *P* = (*F*_o_^2^ + 2*F*_c_^2^)/3
*S* = 1.15	(Δ/σ)_max_ < 0.001
3392 reflections	Δρ_max_ = 1.24 e Å^−^^3^
174 parameters	Δρ_min_ = −0.75 e Å^−^^3^

### catena-Poly[[[diaquabis(N-benzoylglycinato)nickel(II)]-µ-aqua] dihydrate] (2) Special details

**Table:**

Geometry. All esds (except the esd in the dihedral angle between two l.s. planes) are estimated using the full covariance matrix. The cell esds are taken into account individually in the estimation of esds in distances, angles and torsion angles; correlations between esds in cell parameters are only used when they are defined by crystal symmetry. An approximate (isotropic) treatment of cell esds is used for estimating esds involving l.s. planes.

### catena-Poly[[[diaquabis(N-benzoylglycinato)nickel(II)]-µ-aqua] dihydrate] (2) Fractional atomic coordinates and isotropic or equivalent isotropic displacement parameters (Å^2^)

**Table:**

	*x*	*y*	*z*	*U*_iso_*/*U*_eq_	
Ni1	0.500000	0.500000	0.500000	0.01759 (14)	
O1W	0.46631 (6)	0.6995 (4)	0.4192 (3)	0.0285 (4)	
H1WA	0.4671 (12)	0.696 (8)	0.305 (7)	0.058 (14)*	
H1WB	0.4475 (12)	0.690 (7)	0.447 (6)	0.043 (12)*	
O2W	0.500000	0.3793 (4)	0.250000	0.0202 (5)	
H2W	0.4841 (9)	0.322 (6)	0.263 (5)	0.034 (11)*	
O2	0.53585 (5)	0.6801 (3)	0.4257 (2)	0.0268 (4)	
O3	0.55711 (6)	0.7689 (4)	0.6765 (3)	0.0351 (5)	
C8	0.55754 (7)	0.7599 (4)	0.5190 (3)	0.0226 (5)	
C9	0.58529 (8)	0.8493 (5)	0.4233 (4)	0.0300 (6)	
H9A	0.593708	0.753037	0.347050	0.036*	
H9B	0.576431	0.953631	0.354201	0.036*	
N1	0.61229 (6)	0.9234 (4)	0.5260 (4)	0.0309 (5)	
H1	0.6146 (12)	1.056 (8)	0.526 (6)	0.049 (13)*	
O1	0.63274 (9)	0.6289 (4)	0.5816 (5)	0.0642 (10)	
C1	0.63502 (8)	0.8063 (5)	0.5955 (4)	0.0353 (7)	
C2	0.66370 (8)	0.8938 (6)	0.6870 (5)	0.0379 (7)	
C3	0.68705 (12)	0.7700 (8)	0.7561 (6)	0.0591 (12)	
H3	0.683771	0.637691	0.748942	0.071*	
C4	0.71526 (13)	0.8407 (11)	0.8360 (7)	0.0746 (17)	
H4	0.731124	0.755654	0.877935	0.090*	
C5	0.71989 (12)	1.0344 (10)	0.8535 (7)	0.0687 (16)	
H5	0.738643	1.081793	0.908817	0.082*	
C6	0.69707 (12)	1.1556 (8)	0.7899 (6)	0.0602 (12)	
H6	0.700203	1.287520	0.802593	0.072*	
C7	0.66879 (10)	1.0893 (7)	0.7054 (6)	0.0501 (10)	
H7	0.653429	1.176328	0.661677	0.060*	
O3W	0.59732 (7)	0.3249 (4)	0.4678 (4)	0.0389 (6)	
H3WB	0.5916 (14)	0.323 (9)	0.362 (8)	0.076 (19)*	
H3WA	0.611 (2)	0.407 (13)	0.485 (11)	0.12 (3)*	

### catena-Poly[[[diaquabis(N-benzoylglycinato)nickel(II)]-µ-aqua] dihydrate] (2) Atomic displacement parameters (Å^2^)

**Table:**

	*U*^11^	*U*^22^	*U*^33^	*U*^12^	*U*^13^	*U*^23^
Ni1	0.0173 (2)	0.0246 (2)	0.01079 (19)	−0.00141 (17)	−0.00075 (14)	−0.00031 (17)
O1W	0.0269 (10)	0.0380 (12)	0.0204 (9)	0.0067 (9)	−0.0004 (8)	0.0033 (8)
O2W	0.0212 (13)	0.0262 (13)	0.0132 (11)	0.000	−0.0013 (9)	0.000
O2	0.0246 (9)	0.0393 (12)	0.0164 (8)	−0.0116 (8)	−0.0010 (7)	0.0008 (8)
O3	0.0328 (11)	0.0521 (15)	0.0204 (9)	−0.0160 (10)	−0.0010 (8)	−0.0017 (9)
C8	0.0221 (12)	0.0246 (12)	0.0211 (11)	−0.0038 (9)	−0.0002 (9)	0.0029 (10)
C9	0.0283 (14)	0.0373 (16)	0.0242 (12)	−0.0099 (12)	−0.0006 (10)	0.0052 (12)
N1	0.0246 (12)	0.0318 (13)	0.0360 (13)	−0.0082 (10)	−0.0012 (10)	0.0031 (11)
O1	0.067 (2)	0.0349 (15)	0.089 (3)	−0.0063 (14)	−0.0317 (18)	0.0038 (15)
C1	0.0331 (16)	0.0359 (17)	0.0367 (16)	−0.0054 (13)	−0.0027 (13)	0.0024 (13)
C2	0.0294 (15)	0.049 (2)	0.0352 (16)	−0.0032 (14)	−0.0019 (12)	−0.0030 (15)
C3	0.055 (3)	0.057 (3)	0.064 (3)	0.008 (2)	−0.020 (2)	−0.005 (2)
C4	0.047 (3)	0.104 (5)	0.071 (3)	0.018 (3)	−0.025 (2)	−0.006 (3)
C5	0.039 (2)	0.108 (5)	0.058 (3)	−0.020 (3)	−0.010 (2)	−0.014 (3)
C6	0.053 (3)	0.065 (3)	0.062 (3)	−0.024 (2)	−0.009 (2)	−0.004 (2)
C7	0.0386 (19)	0.054 (2)	0.057 (2)	−0.0104 (18)	−0.0091 (17)	−0.002 (2)
O3W	0.0391 (13)	0.0405 (14)	0.0367 (13)	−0.0001 (11)	−0.0027 (10)	−0.0007 (11)

### catena-Poly[[[diaquabis(N-benzoylglycinato)nickel(II)]-µ-aqua] dihydrate] (2) Geometric parameters (Å, º)

**Table:**

Ni1—O2^i^	2.029 (2)	N1—H1	0.93 (5)
Ni1—O2	2.029 (2)	O1—C1	1.240 (5)
Ni1—O1W^i^	2.041 (2)	C1—C2	1.487 (5)
Ni1—O1W	2.041 (2)	C2—C7	1.380 (6)
Ni1—O2W^i^	2.1450 (12)	C2—C3	1.384 (6)
Ni1—O2W	2.1450 (12)	C3—C4	1.386 (7)
O1W—H1WA	0.90 (5)	C3—H3	0.9300
O1W—H1WB	0.81 (5)	C4—C5	1.364 (9)
O2W—H2W	0.77 (4)	C4—H4	0.9300
O2W—H2W^ii^	0.77 (4)	C5—C6	1.342 (8)
O2—C8	1.262 (3)	C5—H5	0.9300
O3—C8	1.246 (3)	C6—C7	1.394 (6)
C8—C9	1.517 (4)	C6—H6	0.9300
C9—N1	1.443 (4)	C7—H7	0.9300
C9—H9A	0.9700	O3W—H3WB	0.86 (6)
C9—H9B	0.9700	O3W—H3WA	0.81 (9)
N1—C1	1.338 (5)		
			
O2^i^—Ni1—O2	180.00 (12)	C8—C9—H9A	108.3
O2^i^—Ni1—O1W^i^	88.72 (10)	N1—C9—H9B	108.3
O2—Ni1—O1W^i^	91.28 (10)	C8—C9—H9B	108.3
O2^i^—Ni1—O1W	91.28 (10)	H9A—C9—H9B	107.4
O2—Ni1—O1W	88.72 (10)	C1—N1—C9	121.4 (3)
O1W^i^—Ni1—O1W	180.0	C1—N1—H1	122 (3)
O2^i^—Ni1—O2W^i^	87.27 (8)	C9—N1—H1	116 (3)
O2—Ni1—O2W^i^	92.73 (8)	O1—C1—N1	121.2 (3)
O1W^i^—Ni1—O2W^i^	89.87 (8)	O1—C1—C2	120.3 (3)
O1W—Ni1—O2W^i^	90.13 (8)	N1—C1—C2	118.5 (3)
O2^i^—Ni1—O2W	92.73 (8)	C7—C2—C3	118.0 (4)
O2—Ni1—O2W	87.27 (8)	C7—C2—C1	124.6 (4)
O1W^i^—Ni1—O2W	90.13 (8)	C3—C2—C1	117.4 (4)
O1W—Ni1—O2W	89.87 (8)	C2—C3—C4	120.8 (5)
O2W^i^—Ni1—O2W	180.0	C2—C3—H3	119.6
Ni1—O1W—H1WA	104 (3)	C4—C3—H3	119.6
Ni1—O1W—H1WB	120 (3)	C5—C4—C3	120.4 (5)
H1WA—O1W—H1WB	109 (4)	C5—C4—H4	119.8
Ni1—O2W—Ni1^ii^	134.02 (15)	C3—C4—H4	119.8
Ni1—O2W—H2W	93 (3)	C6—C5—C4	119.2 (4)
Ni1^ii^—O2W—H2W	111 (3)	C6—C5—H5	120.4
Ni1—O2W—H2W^ii^	111 (3)	C4—C5—H5	120.4
Ni1^ii^—O2W—H2W^ii^	93 (3)	C5—C6—C7	121.8 (5)
H2W—O2W—H2W^ii^	118 (6)	C5—C6—H6	119.1
C8—O2—Ni1	127.02 (18)	C7—C6—H6	119.1
O3—C8—O2	124.9 (3)	C2—C7—C6	119.7 (4)
O3—C8—C9	120.8 (2)	C2—C7—H7	120.2
O2—C8—C9	114.3 (2)	C6—C7—H7	120.2
N1—C9—C8	115.9 (2)	H3WB—O3W—H3WA	110 (7)
N1—C9—H9A	108.3		
			
Ni1—O2—C8—O3	−13.6 (5)	N1—C1—C2—C3	−179.3 (4)
Ni1—O2—C8—C9	166.8 (2)	C7—C2—C3—C4	−2.3 (8)
O3—C8—C9—N1	6.8 (5)	C1—C2—C3—C4	176.8 (5)
O2—C8—C9—N1	−173.5 (3)	C2—C3—C4—C5	2.5 (9)
C8—C9—N1—C1	78.0 (4)	C3—C4—C5—C6	−1.1 (9)
C9—N1—C1—O1	−3.9 (6)	C4—C5—C6—C7	−0.4 (9)
C9—N1—C1—C2	174.6 (3)	C3—C2—C7—C6	0.8 (7)
O1—C1—C2—C7	178.3 (4)	C1—C2—C7—C6	−178.2 (4)
N1—C1—C2—C7	−0.2 (6)	C5—C6—C7—C2	0.5 (8)
O1—C1—C2—C3	−0.7 (6)		

Symmetry codes: (i) −*x*+1, −*y*+1, −*z*+1; (ii) −*x*+1, *y*, −*z*+1/2.

### catena-Poly[[[diaquabis(N-benzoylglycinato)nickel(II)]-µ-aqua] dihydrate] (2) Hydrogen-bond geometry (Å, º)

**Table:**

*D*—H···*A*	*D*—H	H···*A*	*D*···*A*	*D*—H···*A*
O1*W*—H1*WA*···O2^ii^	0.90 (5)	1.82 (5)	2.726 (3)	174 (5)
O1*W*—H1*WB*···O3*W*^i^	0.81 (5)	1.97 (5)	2.783 (4)	176 (5)
O2*W*—H2*W*···O3^i^	0.77 (4)	1.88 (4)	2.634 (3)	167 (4)
C9—H9*B*···O3^iii^	0.97	2.50	3.465 (4)	176
N1—H1···O3*W*^iv^	0.93 (5)	2.04 (5)	2.888 (4)	151 (4)
O3*W*—H3*WB*···O3^v^	0.86 (6)	2.10 (6)	2.858 (4)	147 (5)
O3*W*—H3*WA*···O1	0.81 (9)	1.92 (9)	2.697 (4)	159 (8)

Symmetry codes: (i) −*x*+1, −*y*+1, −*z*+1; (ii) −*x*+1, *y*, −*z*+1/2; (iii) *x*, −*y*+2, *z*−1/2; (iv) *x*, *y*+1, *z*; (v) *x*, −*y*+1, *z*−1/2.
